# Biomarkers and cell-based models to predict the outcome of neoadjuvant therapy for rectal cancer patients

**DOI:** 10.1186/s40364-021-00313-9

**Published:** 2021-07-28

**Authors:** Aylin Alkan, Tobias Hofving, Eva Angenete, Ulf Yrlid

**Affiliations:** 1 Department of Surgery, Institute of Clinical Sciences, Sahlgrenska Academy, University of Gothenburg, Gothenburg, Sweden; 2 Region Västra Götaland, Department of Surgery, SSORG - Scandinavian Surgical Outcomes Research Group, Sahlgrenska University Hospital, Gothenburg, Sweden; 3 Department of Microbiology and Immunology, Sahlgrenska Academy, University of Gothenburg, Gothenburg, Sweden

**Keywords:** Rectal cancer, Biomarkers, Neoadjuvant therapy, Microenvironment, Patient-derived xenografts, Patient-derived organoids

## Abstract

Rectal cancer constitutes approximately one-third of all colorectal cancers and contributes to considerable mortality globally. In contrast to colon cancer, the standard treatment for localized rectal cancer often involves neoadjuvant chemoradiotherapy. Tumour response rates to treatment show substantial inter-patient heterogeneity, indicating a need for treatment stratification. Consequently researchers have attempted to establish new means for predicting tumour response in order to assist in treatment decisions. In this review we have summarized published findings regarding potential biomarkers to predict neoadjuvant treatment response for rectal cancer tumours. In addition, we describe cell-based models that can be utilized both for treatment prediction and for studying the complex mechanisms involved.

## Introduction

Rectal cancers make up approximately one-third of colorectal cancers (CRCs) and contribute to a high cancer-related mortality in both men and women globally. Tumours in the rectum share molecular aspects with tumours in the colon [1], though treatment strategies differ. Rectal cancers are more often treated with neoadjuvant treatment including (chemo)radiotherapy to reduce the risk of local recurrence. Neoadjuvant treatment may also increase the chances for a sphincter sparing procedure [2, 3]. 5-Fluorouracil (5-FU), oxaliplatin and capecitabine are commonly used chemotherapeutic agents [4, 5]. However, the response to neoadjuvant (chemo)radiotherapy varies considerably among rectal cancer patients. Approximately 10–20% of patients with locally advanced rectal cancer (LARC) display a complete pathological response to neoadjuvant therapy and could avoid surgery (watch and wait) [6]. Finding tools to identify these patients prior to treatment has become an area interest for researchers and clinicians alike.

Little is known about the underlying mechanisms governing tumour response to neoadjuvant chemoradiotherapy . Clinical and radiological factors that may play a role have been identified, such as size of the tumour, TNM stage, radiation dose and fractioning, as well as the time period between neoadjuvant chemoradiotherapy and surgery [7]. However, clinical and radiological parameters have so far only reached a limited specificity and sensitivity [8–10].

In recent years, novel methods to improve the prediction of sensitivity to therapies have been developed, such as proposed novel biomarkers [8]. While many of these biomarkers show potential, there is an urgent need to further validate them to facilitate their transition into the clinical setting. Current and emerging cell-based experimental models, such as mouse xenografts and tumouroid cultures, have been used to aid personalized medicine. For rectal cancer, such models have been used both to predict patient tumour response to neoadjuvant therapy and to serve as platforms for investigating functional mechanisms. In this review, we summarize emerging biomarkers (Table 1) and cell-based models that have been proposed to predict neoadjuvant treatment response in rectal cancer.

**Table 1:** Biomarkers suggested to predict neoadjuvant treatment response in rectal cancer

Category	Type	Parameter
**Molecular genetic markers**	DNA mismatch repair [11–21]	dMMR, MSI, MSI-H
	MAPK/ERK and PI3K/AKT/mTOR pathways [22–37]	EGF, EGFR, VEGF, RAS, KRAS, BRAF, PTEN, NF1
	Tumour suppressors and oncogenes [38–46]	TP53, XIAP, TCF4
	Transcriptome/Epigenome [47–71]	Transcriptomic and epigenetic signatures, miR-130a, miR-487a-3p, *TIMP3* methylation
**Immunological markers**	Blood-based immunological markers [72–86]	P/L ratio, N/L ratio, cytokines, C-reactive protein
	Tissue-based immunological markers [87–119]	LNR, TILs, PD-1 and PD-L1
**Other biomarkers**	Blood-based cancer markers [120–139]	CTCs, cfDNA, ctDNA, CEA
	Gut microbiota [140–144]	*Fusobacteria, Bacteroidales, Duodenibacillus massiliensis*

*Abbreviations*: *dMMR* Mismatch repair deficient, *MSI* Mismatch repair instable, *MSI-H* Mismatch repair instable-high, *EGF* Epidermal growth factor, *EGFR* Epidermal growth factor receptor, *VEGF* Vascular endothelial growth factor, *RAS* Rat sarcoma, *KRAS* Kirsten rat sarcoma, *BRAF* V-raf murine sarcoma viral oncogene homolog B1, *PTEN* Phosphatase and tensin homolog, *NF1* Neurofibromin 1, *TP53* Tumour protein p53, *XIAP* X-linked inhibitor of apoptosis protein, *TCF4* Transcription factor 4, *TIMP3* Tissue inhibitor of matrix metalloproteinases 3, *P/L ratio* Platelet-to-lymphocyte ratio, *N/L ratio* Neutrophil-to-lymphocyte ratio, *LNR* Lymph node ratio, *TILs* Tumour infiltrating lymphocytes, *PD-1* Programmed cell death protein 1, *PD-L1* Programmed death-ligand 1, *CTCs* Circulating tumour cells, *cfDNA* Cell-free DNA, *ctDNA* Circulating tumour DNA, *CEA* Carcinoembryonic antigen

## Molecular and genetic markers in rectal cancer

The outstanding complexity of molecular cell signalling is disrupted in cancer. The dysregulations that result in cancer growth originate in changes to the DNA. While the exact mutational progression and tumour development differ from patient to patient, many cancer types share common features. Rectal tumours can be sub-grouped based on genetic traits such as gene mutations, RNA expression and epigenetic modifications [1]. These genetic traits can also be utilized in attempts to identify parameters that can stratify patient tumours prior to neoadjuvant therapy in order to identify which tumours that will respond favourably.

### DNA mismatch repair

DNA mismatch repair deficiency is a well-established biomarker in colorectal cancer. Tumours are typically classified as either MMR proficient (pMMR) or MMR deficient (dMMR), where the latter can lead to microsatellite instability (MSI). MSI accounts for about 15% of all CRC [11]. Due to their increased tumour mutational burden, MSI-H (high) colon cancers are associated with both more extensive immune cell infiltration and anti-tumour immune responses [12]. Why these tumours progress despite this is probably due to check-point inhibition [13]. Consequently, treatment with check-point inhibitors has been efficacious and is now often offered to MSI-H colorectal cancer patients with resistance to first line of treatment [14]. However, using microsatellite stability as a predictor of neoadjuvant chemoradiation sensitivity for rectal cancer patients remains controversial. Some reports have suggested that MSI is associated with better response [15–17], while others have shown lack of better clinical outcome [18–20]. A recent analysis of 5086 patients (of which 4450 were MSI-negative and 636 MSI-positive) showed that MSI-positive tumours were associated with a higher tumour grade and resulted in fewer pathological complete responses after neoadjuvant chemoradiation [17]. MSI-H was reported not be a tool for the prediction of overall survival of stage II and III rectal cancer patients [18]. However, MSI-H rectal cancers patients in another study were found to correlate with an adverse prognosis [19]. In a recent meta-analysis surveying nine published studies, no association between patients achieving pathological complete response following neoadjuvant chemoradiotherapy and their MSI status could be found [20]. Hence, while there is a solid prognostic value of MSI in colorectal cancer, the predictive potential in rectal cancer is not clear [21]. It is conceivable that MSI-status could be used as a future tool to aid treatment decisions for rectal cancer patients if it is further validated. It is, however, important to note that the vast majority of rectal cancers (> 90%) are not MSI-H, and utilization of MSI-H as a marker will only be applicable to a subset of patients.

For rectal cancers, mismatch repair does not seem to fulfil the criteria for a useful predictive marker due to its applicability to the majority of patients.

### The MAPK/ERK and PI3K/AKT/mTOR signaling pathways

Both the MAPK/ERK and the PI3K/AKT/mTOR signaling pathways are involved in many and diverse intracellular functions within the cell and can among other things lead to proliferation, differentiation and apoptosis [22, 23]. These two pathways have in common that they can be initiated by tyrosine kinase receptor signalling, such as by the epidermal growth factor receptor (EGFR). Mutations in both the MAPK/ERK pathway (e.g. *BRAF*, *KRAS*, *NRAS*, *ERBB2*, and *ERBB3*) and the PI3K/AKT/mTOR pathway (e.g. *PIK3CA*, *IGF2*, *PTEN*, and *PIK3R1*) are frequent in rectal cancer [1]. The prognostic and predictive values of the expression of EGFR and mutational status of several proteins involved in these signalling pathways have been investigated.

In pre-treatment samples from patients with rectal adenocarcinoma, higher gene expression of *EGFR* and *VEGF*, the latter another tyrosine receptor ligand capable of activating the pathways, has been observed in non-responders compared to responders to chemoradiotherapy [24]. In addition, a positive immunohistochemical EGFR staining of the tumours was associated with lower pathological complete response rates after treatment and worse disease-free survival [25]. Cetuximab, a monoclonal antibody therapy targeting EGFR has been shown efficacious in treating CRC [26]. According to a study with 57 LARC patients treated with cetuximab-based chemoradiotherapy, there was no correlation between mutations in *KRAS*, *BRAF* or *PTEN* and tumour response or 3-year disease-free survival rate [27].

A more recent study with LARC patients showed potential mutational differences between complete or poor responders [28]. Studies correlating mutations in genes involved in canonical MAPK/ERK pathway signalling to neoadjuvant therapy response have reported varying results. Several studies have failed to correlate treatment outcome to *KRAS* mutational status [29–31]. However, in one study with 229 LARC patients, an association with poor treatment response was found [32], and mutations of specific *KRAS* codons have been found to be associated with varying treatment response [33, 34].

Inhibiting the PI3K/AKT/mTOR pathway has been shown by several authors to increase the sensitivity to radiotherapy for various cancers (reviewed in [35]). Rapamycin is an mTOR inhibitor which, when administered to rectal cancer patients 1 week before as well as during radiotherapy treatment, reduced the metabolic activity of tumours without affecting the tumour response to treatment [36]. One study concluded that while mutations in *PTEN* and *NF1* were common, their mutational status did not affect response to treatment [28].

Epiregulin is in the EGF family of proteins and is an EGFR agonist. In one study, epiregulin was examined with immunohistochemistry in 172 rectal cancer biopsies collected before neoadjuvant chemoradiotherapy [37]. The study showed that higher epiregulin expression prior to treatment was significantly associated with better disease-specific survival, locoregional recurrence-free survival, and metastasis-free outcome, thus suggesting that epiregulin could be a potential therapeutic target and predictive marker.

The expression level of EGFR and the activity of intracellular signalling components in rectal cancers influence the odds for a complete response to current treatments. However, additional studies are still needed to clarify the role of EGFR and the predictive potential of the MAPK/AKT and PI3K/AKT/mTOR pathways in rectal cancer.

### Other tumour suppressors and oncogenes

The use of tumour suppressor p53 in evaluating neoadjuvant chemoradiotherapy sensitivity of rectal cancer tumours remains controversial. For instance, it has been reported that increased nuclear expression of p53 is associated with radiotherapy treatment resistance [38]. Others have suggested that p53 expression has a role in predicting a favorable response to neoadjuvant chemoradiotherapy [39, 40]. Meta-analysis of data from 1830 rectal cancer patients showed that favorable clinical response to neoadjuvant chemoradiation was observed in wild type p53 [41], and a smaller study found that p53 expression after treatment was increased in 6 out of 9 non-responders compared to p53 expression prior to treatment [42].

Increased levels of X-linked inhibitor of apoptosis protein (XIAP) in rectal cancer cells have been found to mediate resistance to neoadjuvant chemoradiotherapy [43]. Another example of a promising target gene is T cell-specific factor 4 (*TCF4*) which has a differential expression profile in responsive and resistant tumours [44]. TCF4 (also known as TCF7L2) is a transcription factor that is involved in the Wnt signalling pathway, and it has been found that constitutive activation of β-catenin/TCF4 can promote colon cancer development [45]. One study suggested that low protein expression of TCF4 could be used as a prediction marker for good response to treatment and was correlated to better clinical outcomes in terms of 5-year overall survival and 5-year disease-free survival [46]. In summary, although several studies have investigated the mutational status of tumour suppressors and oncogenes and their expression in rectal cancer, none are yet used clinically to personalize treatments.

### Transcriptomic and epigenetic signatures

Tumour responsiveness to neoadjuvant therapy is complex, and single genes, transcripts or proteins may have limited impact. Addressing this, several studies have instead attempted to establish RNA transcription or epigenetic signatures of rectal pre-treatment biopsies with the aim of better reflecting and predicting tumour response to neoadjuvant therapy.

Several mRNA transcription signatures, in particular using microarray-based platforms, have been suggested in various studies [47–57]. The impact of these studies has so far been limited. In 2015, Lopes-Ramos et al. in addition to developing their own mRNA gene signature, evaluated how signatures published prior to the study fared in their own cohort [48]. The studies involved between 30 and 62 cases and identified signatures of 4–95 differentially expressed genes. The results were largely disappointing, with all evaluated signatures performing poorly compared to current imaging and clinical parameters. Other evaluations of published microarray based gene signatures have shown similar issues, with signatures having either low sensitivity or selectivity [51]. More recently, another microarray study by Guo et al. investigated a somewhat larger patient cohort (42 in training cohort, 33 for validation) showed higher accuracy, around 90% [49].

Attention has also turned to non-coding RNAs and epigenetics. Investigated non-coding RNAs have mainly involved micro-RNA (miRNA). Although non-coding, these transcripts contribute to the vast complexity of regulating mRNA function and protein translation [58] and carry the benefit of being measurable in blood [59]. Reviewed by Pettit et al. [60], 12 studies analyzing the expression of miRNA, using platforms such as TaqMan microRNA, miScript assay, and Agilent SurePrint Technology, in patient biopsies related to tumour treatment response, have identified very diverse sets of miRNAs correlating to pathological complete response of the tumour. Many of the miRNAs identified in these studies involve the DNA damage response, the cell-cycle, and apoptosis [60]. In addition, other studies have suggested single miRNAs, such as miRNA-130a [61] and miR-487a-3p [62], as well as combinations of various miRNA transcripts [63, 64]. Long non-coding RNAs (lncRNAs) are RNA transcripts longer than miRNAs, typically more than 200 nucleotides long. In the context of predicting response to neoadjuvant therapy in rectal tumours, this class has also been studied, with several identified candidates and signatures [65–67].

Epigenetics, and in particular DNA-methylation, has also been explored. One example is methylation of the *TIMP3* gene, which was found to significantly correlate to tumour regression grade after neoadjuvant therapy [68]. Several other studies have studied methylation status of genes in correlation to tumour response, which has been reviewed for rectal [69] and colorectal tumours [70]. A recent study by Canto et al. analyzed CpG methylation of 32 rectal tumour biopsies prior to treatment. From these, they proposed a classifier based on three sites with differentially methylated CpGs (linked to *OBSL1*, *GPR1*, and *INSIG1*). The classifier was then validated with pyrosequencing in a mixed cohort of 77 LARCs and showed 93.8% sensitivity and 67.3% specificity [71].

## Immunological markers

While immunotherapy in rectal cancer has yet to show the same success as it has for several other cancer diagnoses, the importance of immune infiltration in rectal cancer tumours is undoubted, and reflected in the proposed predictive value of many immunological parameters.

### Blood-based immunological markers

#### Immune cell ratios

Neutrophil-to-lymphocyte (N/L) and platelet-to-lymphocyte (P/L) ratios in blood have been used for prognostication of gastrointestinal cancers. If and how these cells could promote tumour progression has not yet been established [72, 73]. Lymphocytes, neutrophils, and platelets are generally routinely quantified in blood-samples at the clinic. Hence, the correlation of these ratios to the outcome of neoadjuvant chemoradiotherapy treatment for rectal cancer patients have been assessed in several studies.

A study investigating the N/L ratio before and after neoadjuvant chemoradiotherapy treatment in patients with rectal cancer found that the N/L ratio prior to treatment was a predictor of poor tumour treatment response [74]. Elevated N/L ratio after the treatment was, however, associated with a worse outcome. Another study looked at the N/L ratio of tumours post-neoadjuvant treatment but prior to surgery, and found that poor responders had a significantly higher value of N/L evaluated after neoadjuvant therapy compared to good responders [75]. The cut-off value of the N/L ratio was determined to 4.5 for the predictive value of poor response. In agreement, another study including data from patients with rectal cancer receiving neoadjuvant concurrent chemoradiotherapy, showed that patients with high N/L ratio had significantly worse 5-year disease-free survival as well as overall survival [76]. Moreover, patients with high P/L ratio had a significantly worse 5-year disease-free survival, and stage II and III patients with high N/L ratio had worse 5-year disease-free survival and overall survival. Yet, another study didn’t find any correlation with N/L or P/L ratios and survival [77], and a recently published retrospective study of 1052 rectal cancer patients [78] showed that an N/L ratio above 3.1 was significantly associated with increased overall survival.

The role for these immune cell ratios for prognostication is disputed, studies investigating immune cell ratios prior to treatment are few and have reached limited conclusions. However, considering the practicality of blood-based biomarkers in sample-obtaining and processing, additional studies may be warranted.

#### Cytokine levels

Cytokines are mainly secreted by leukocytes but other cells also produce these factors which drive and modulate immune responses [79]. As multiparametric approaches for measurements have become available, various sets of cytokines, chemokines and soluble ligands have been assessed in blood samples from rectal cancer patients and correlated with treatment response.

For example, a study using a bead-based multiplex approach quantified the abundance of ten different cytokines and chemokines in plasma from patients before and after chemoradiotherapy [80]. They found that the levels of TNF-α and IL-6 were significantly higher after treatment in non-responders compared to responders. In addition, a significant decrease in the levels of soluble CD40L pre-treatment and CCL-5 after treatment was detected in responders [80]. In another study, neopterin, which is expressed by macrophages in response to IFN-γ stimulation, was evaluated in patients treated with neoadjuvant chemoradiotherapy [81]. High serum level of neopterin (cut-off 3 μg/l) prior to treatment was shown to be a predictor of poorer overall and relapse-free survival after chemoradiotherapy. Transforming growth factor beta 1 (TGF-β1) has been indicated as a marker for metastases, as the level of TGF-β1 was higher in patients with metastases at primary diagnosis compared to patients without metastases [82]. Moreover, lower level of total TGF-β1 was found in plasma samples from patients who developed metastases later.

These studies indicate that cytokine levels in patient serum could be useful, but analyses of multiple cytokines are most likely needed to be combined for a more general clinical applicability, and any such indicator must be further validated.

#### C-reactive protein

The association of C-reactive protein with the prediction of survival and treatment response in rectal cancer has been examined in several prospective studies [83–85]. Elevated CRP level was identified as an independently significant predictive factor for poor disease-free survival in patients with rectal cancer treated by chemoradiotherapy [83]. Also, elevated CRP was found associated with poor outcomes after chemoradiotherapy and surgery for rectal cancer. In addition to that, CRP expression was observed to be significantly higher in nonresponders than in responders [84]. In another study, they found that the elevated CRP group had significantly lower 5-year disease-free survival and cancer-specific survival [85]. A recently published study analyzed 86 patients with rectal cancer who received preoperative chemoradiotherapy and they verified lymphocyte-CRP ratio as a predictive biomarker [86]. They found that post-treatment lymphocyte-CRP ratio status was not correlated with overall survival. However, low pre-treatment lymphocyte-CRP ratio was significantly associated with shorter recurrence-free survival and overall survival.

The findings suggest that CRP level shows prognostic significance in rectal cancer patients. It may be used to identify patients who need additional therapy to improve tailored treatment.

### Tissue-based immunological markers

The type, quantity and location of tumour-infiltrating lymphocytes (TILs) have been thoroughly investigated in pre- and post-treatment tissue biopsies of rectal cancer with the aim to predict the response to treatment. Recently, lymph-node ratio (LNR) has emerged as a prognostic tool and is known as a predictive marker for survival in rectal cancer.

#### Lymph node ratio

The lymph node ratio (LNR), the ratio between the number of surgically removed metastatic and healthy lymph nodes, has been used as a prognostic marker in rectal cancer. Studies have shown that the LNR is an independent biomarker for survival in rectal cancer [87–90]. In one of the studies, 131 patients with rectal cancer were separated into two different groups based on the LNR value (≤0.2 [*n* = 86], > 0.2 [*n* = 45]) [87]. A high LNR value was significantly correlated with worse disease-free and overall survival in node-positive patients. In another study there was no significant difference in LNR value in patients with stage III rectal cancer regarding overall as well as disease-free survival [91]. However, a recent systematic review using meta analysis has reported that a high LNR value is highly correlated with inferior overall survival and disease-free survival [90]. Although many studies have shown that LNR could be used as a prognostic tool for survival of rectal cancer patients, assessment of whether neoadjuvant chemo-radiotherapy impacts the lymph node ratio in patients with rectal cancer and LNR value in patients treated with neoadjuvant therapy affects the outcome of treatments, needs further evaluation.

#### CD8^+^ T cells

Having high amounts of CD8^+^ T cells both prior to neoadjuvant treatment and post-treatment have been shown in most [92–99] but not all studies [100, 101] to be favorable factors for tumour response in rectal cancer. In 2011, Yasuda and colleagues reported a histochemical assessment of advanced rectal cancers showing that higher densities of CD8^+^ as well as CD4^+^ TILs in pre-treatment biopsies were correlated to a good tumour response after chemoradiotherapy in terms of reduced tumour size and histological grade [92]. Another study similarly found that low densities of CD8^+^ TILs both in pre-treatment samples and in post-treatment samples were associated with a poor treatment outcome [93]. Yet other studies have shown that the density of CD8^+^ T cells is significantly increased in post-treatment samples compared to pre-treatment [93, 94]. Densities of CD3^+^ and CD8^+^ TILs in pre-neoadjuvant chemoradiotherapy samples were shown to be associated with good response to the treatment [95, 96]. In addition, densities of CD3^+^ and CD8^+^ TILs in post-neoadjuvant chemoradiotherapy samples were found to be higher compared to their corresponding pre-treatment samples [95, 96], and the number of CD8^+^ T cells and T cells expressing granzyme B (GrzB^+^) (which suggests cytotoxic potential) in tumour stroma is increased in rectal cancer after neoadjuvant chemoradiotherapy [97]. Interestingly, the total numbers of both CD3^+^ and CD8^+^ T cells in tumour epithelium as well as in tumour stroma were found to be significantly lower after treatment. The increase in number of CD8^+^/GrzB^+^ cytotoxic T cells in post-treatment tumour samples was also associated with a lower likelihood of local recurrence and higher regression grade, suggesting that CD8^+^/GrzB^+^ T cells may promote a better outcome and contribute to local tumour control. Indeed, a high density of CD8^+^ TILs has been suggested as a useful biomarker for a better prognosis of rectal cancers [94, 98]. A recent expression analysis has shown that increased diversity of T cell receptor repertoires before and after neoadjuvant chemoradiotherapy are correlated with better recurrence-free survival [99].

A forthcoming histochemical assessment of T cell infiltration termed ‘immunoscore’ has been extensively validated and is a powerful prognostic tool for patients with colon cancer. Its capacity has indeed been demonstrated to surpass that of classical TNM scoring [102, 103]. Immunoscore is based on densities of CD3^+^ and CD8^+^ T cells in the invasive margin and in the tumour itself. From this, tumours are stratified into categories where for example the category of tumours with high densities of T cells at both locations have the most favorable prognosis. The prognostic applicability of immunoscore for rectal cancer patients not receiving chemoradiotherapy has been confirmed [104]. In a recently published study, a biopsy-adapted immunoscore (ISB) was assessed in LARC patients to determine if this could be used to predict response to chemoradiotherapy [105]. ISB was found to be positively correlated with histological response, and ISB high patients had lower risk of death or relapse compared to patients that were ISB low. In addition, in a cohort of “watch and wait” patients not receiving surgery after chemoradiotherapy the patients in the ISB high group (*N* = 17) showed no relapse during the study.

**Fig. 1:**
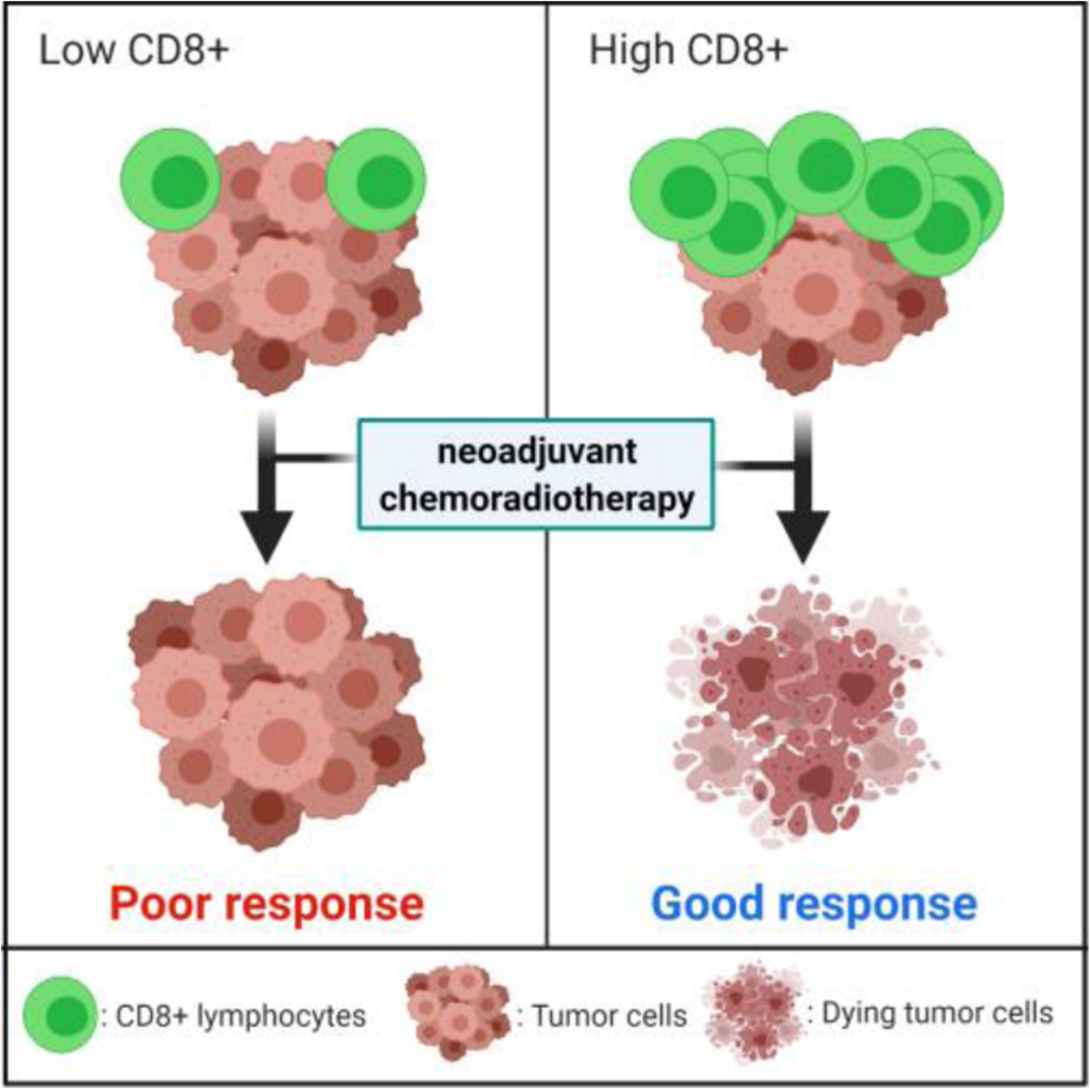
The abundance of CD8+ TILs in the rectal tumour microenvironment before neoadjuvant chemoradiotherapy has been correlated to patient prognosis. Higher expression of CD8^+^ T cells pre-treatment leads to a more favorable patient prognosis

These studies have shown that the extent of T cell tumour infiltrates, with emphasis on CD8^+^ TILs, constitute an important variable for predicting the response to neoadjuvant chemoradiotherapy (Fig. 1). It may also serve as an important prognostic tool for survival and a potential selection criteria for inclusion in a “watch and wait” regimen.

#### FoxP3^+^ T cells

CD4^+^ T cells can have both anti- and pro-tumourigenic capacity. In particular regulatory CD4^+^ T cells (often defined by FoxP3 expression) have, in this regard, received extra attention. These T cells are targets for immune checkpoint blockade treatment with anti-CTLA4, and a low density of FoxP3^+^ TILs is associated with favorable prognosis in most solid tumours [106, 107]. Some studies have, however, shown that increased levels of infiltrating regulatory T cells are linked to a favorable prognosis for patients with colorectal cancer [108, 109]. A study comparing biopsies from rectal cancer patients pre- and post-chemoradiotherapy showed that stromal FoxP3^+^ TILs remained stable while stromal CD8^+^ T cells increased [110]. High post-treatment stromal CD8^+^ TILs numbers were found strongly correlated with better prognosis, and a high pre-treatment intraepithelial CD8/FoxP3 ratio was found to be a predictor of tumour regression. Neoadjuvant chemoradiotherapy has also been reported to increase the density of CD4^+^ TILs but with maintained expression of CTLA-4 and densities of FoxP3^+^ [94]. An immunohistochemical analysis of surgical specimens from LARC patients found that low stromal FoxP3^+^ cell density after radiotherapy was associated with a favorable regression grade [111]. However, when biopsies from these patients were analyzed prior to therapy, neither FoxP3^+^ nor CD8^+^ T cells correlated with treatment outcome [100]. Also, another immunohistochemical analysis showed instead, that a high post-radiotherapy FoxP3^+^ TIL density correlated with better progression-free survival [101]. In the same study no correlation of pretreatment CD8^+^ TILs and survival was detected, while a decrease in CD8/FoxP3 ratio after treatment predicted better overall and progression-free survival. A more recent study comparing pre-treatment and biopsy material obtained from rectal tumours 7 days after irradiation showed that a high density of CD4^+^ and FoxP3^+^ cells pre-treatment was significantly associated with tumour shrinkage, and that this link was most pronounced in the 7 day biopsies [112]. The location of CD8^+^ and FoxP3^+^ T cells in relation to each other as well as the distance from the tumour cells (stromal vs intra-tumoural) could reflect functionality. This might help to explain some of the differences obtained in the correlations of patient survival and histochemical analyses of TILs in rectal cancers, as the latter have not been enumerated in the stromal and intra-tumoural areas separately in all studies. Indeed, a study investigating the distance between FoxP3^+^ and CD8^+^ T cells pre- and post- chemoradiotherapy in the stroma and the tumour has also indicated that a short distance between the two cell types in the tumour epithelium is associated with a favorable prognosis, and that the opposite was observed in the stromal compartment [113]. In a recent follow-up study the distance of the epithelial-stromal to CD8^+^ and to FoxP3^+^ cells were assessed and suggested to be a more precise prognostic tool than mere cell counting of the two cell types [114].

These studies showed that the density of CD4+ T cells might play an important role in the tumour microenvironment as another subset of tumour-infiltrating lymphocytes. However, as there are some controversial results, further investigation on this specific T cell subtype is required to evaluate the clinical relevance in neoadjuvant chemoradiotherapy in rectal cancer.

#### PD-1/PD-L1

PD-L1 (also known as CD274) is a cell-surface protein that is frequently upregulated in cancer cells. It binds to the PD1 receptor on activated T cells to transmit inhibitory signals and as such prevents cell killing. This ligand/receptor interaction can be blocked by immune checkpoint inhibitor drugs. Consequently PD-L1 expression in rectal cancers after treatment has also been assessed together with T cell stainings. An increase in stromal immune PD-L1 expression after chemoradiotherapy has been reported [115]. This increase was significantly associated with a higher CD8^+^ T cell density in the tumour before the treatment and a higher CD8^+^ T cell density in the stromal compartment after treatment. It has also been reported that the density of CD8^+^ TILs and PD-L1 expression in the tumour was significantly increased after neoadjuvant chemoradiotherapy treatment [116]. In addition, this study showed that patients with higher PD-L1 expression and higher CD8^+^ TILs both pre- and post-treatment had improved disease-free survival. In a patient cohort of colorectal tumours not receiving neoadjuvant therapy, PD-L1 expression was reported to be associated with a poorer prognosis for patients with rectal tumours, but not for patients with colon tumours [117].. Also, immunostainings of PD-L1 have shown that high levels of PD-L1 before and after chemoradiotherapy were found to be associated with poorer disease-free survival and overall survival [118]. Finally, low expression of PD-L1 pre-treatment has been used as a negative prognostic marker of the overall survival for rectal cancer patients [119].

In summary, PD-L1 appears to be induced by chemoradiotherapy, but how to interpret the levels of PD-L1 staining before and after treatment for prediction and prognostication is still unclear. Since PD-1 functions as an immune checkpoint, PD-1 and its ligand PD-L1 are promising targets for immunotherapy. Evaluation of PD-L1 in the tumour microenvironment could be valuable for predicting the response to neoadjuvant chemoradiotherapy.

## Other biomarkers

### Circulating tumour cells, cell-free tumour DNA and circulating tumour DNA

Circulating tumour cells (CTCs) are potentially promising biomarkers to predict response to treatment, prognosticate and predict recurrence in various types of cancers, including colorectal cancer [120]. Most research has been performed in CRC tumours, while a few studies demonstrate results only for rectal tumours. Epithelial cell adhesion molecule (EPCAM)-magnetic bead-based enrichment of epithelial cells (which includes tumour cells) combined with cytometric identification (CellSearch system) were used in a study of patients with rectal cancer [121]. CTCs were detected in all patients with rectal cancer but not in healthy controls. A significant difference in the levels of CTC before and after treatment was also observed between responders and non-responders. In addition, metastatic rectal cancer patients had significantly higher levels of CTCs compared to patients with stage II-III rectal cancer or recurrence [121]. The CellSearch system has also been used for LARC patients where the level of CTCs in blood samples from responders after neoadjuvant treatment was decreased while there was no significant change in non-responders [122]. Moreover, in another study the CTC count pre-treatment was found to be significantly higher in responders than in non-responders [123]. According to the results of this study, after the neoadjuvant chemoradiotherapy, the counts of CTCs in responders were significantly lower than among non-responders.

Cell-free DNA (cfDNA) is another potential biomarker in rectal cancer. The concentration of cell-free DNA in plasma and whether it include *KRAS* mutations and/or O6-methylguanine-DNA methyltransferase (MGMT) promoter methylation has been determined in LARC patients [124]. The concentrations of cfDNA were higher in rectal cancer patients compared to healthy controls. After chemoradiotherapy the frequency of *KRAS* mutations was lower in both poor and good responders. Moreover, the status of MGMT methylation in baseline cfDNA was significantly higher in responders compared to non-responders. Thus CTCs and cfDNA hold a certain promise as useful tools to evaluate the effect of chemoradiotherapy in patients with rectal cancer.

Moreover, circulating tumour DNA (ctDNA) provides important information for the diagnosis of several malignant tumours [125]. ctDNA might be a specific biomarker for diagnosis of rectal cancer and to predict treatment response for rectal cancer patients [126]. In a study including plasma samples from 159 LARC patients, ctDNA was found in 77% of plasma samples before treatment, in 8% during chemoradiotherapy and in 12% at post-treatment [127]. Another study including serial plasma collection from 119 LARC patients, who received neoadjuvant chemoradiotherapy showed that mutations of *TP53* and *APC* genes in pre-treatment samples were detected and these mutations were negatively correlated with the response to neoadjuvant chemoradiotherapy [128]. In a recent study, among 29 patients with LARC treated with neoadjuvant chemoradiation, patients with undetectable pre-operative ctDNA had a favorable surgical outcome, and the study confirmed that detectable postoperative ctDNA was associated with worse recurrence-free survival [129]. All these studies evaluated the potential role of ctDNA as a biomarker to predict the treatment outcomes in locally advanced rectal cancer. There is, however, still a need for more studies to evaluate the potential role of ctDNA to guide patient selection for treatment strategies.

### CEA levels in blood

Alterations in carcinoembryonic antigen (CEA) levels following neoadjuvant chemoradiotherapy has been extensively studied (reviewed in [8, 130]). However there is as yet no validated cut-off value for CEA levels with adequate specificity and sensitivity [8]. Studies including rectal cancer tumours have suggested that patients with pre-treatment CEA levels lower than 2.5 ng/ml have overall better pathological complete response rates compared to those with higher values [131, 132]. Additional research groups have used different cut-off values, and suggested that pretreatment CEA levels could be used as a prediction tool for tumour response [133–135]. In contrast, others have not found any correlation between the pretreatment levels of CEA and tumour regression [136, 137] but instead found a relationship with post-treatment CEA levels lower than 5 ng/ml and pathological complete response, longer disease-free survival and longer overall survival [136]. This has been supported in that a significant correlation between post treatment CEA levels (cut-off 2.61 ng/ml) and pathological complete response has also been reported [137]. Patients with a normalized post-treatment CEA level showed increased overall survival and disease-free survival compared to patients with elevated CEA level [138]. Moreover, higher post-treatment CEA was found as an unfavorable prognostic marker for overall survival in LARC patients with elevated pre-treatment CEA. In a recent study, a combination of imaging and CEA levels was evaluated before and after chemoradiotherapy [139]. They found that this integrated model significantly improved prediction accuracy. Hence CEA could be a useful biomarker for prognosis and for monitoring response to treatment of rectal cancer, but cut-off values are still debated, and perhaps a combination of markers is a solution.

### Gut microbiota

The gut microbiota has been identified as a potentially important factor in how cancer respond to therapies [140], and there have been attempts to modulate it to yield more favorable outcomes [140, 141]. A few recent studies have assessed if and how the microbiome may be associated with rectal cancer response to neoadjuvant therapy. A genomic and transcriptomic study investigating rectal tumour biopsies prior to neoadjuvant therapy did not identify any genetic predictors, but by using the RNAseq data to investigate microorganisms in the biopsies, the authors concluded that a greater presence of *Fusobacteria* correlated with a worse pathological response [142]. Another study, instead looking at fecal samples from patients with rectal tumours, found a change in microbiome composition comparing samples prior to and post-treatment [143]. From pre-treatment biopsies they were also able to construct a classifier to predict treatment outcome of neoadjuvant therapy based on ten genera variables, where *Dorea, Anaerostipes* and *Clostridium XVIII* were biomarkers of neoadjuvant chemoradiotherapy responders, and *Eisenbergiella, Granulicatella* and *Ralstonia* were biomarkers of neoadjuvant chemoradiotherapy non-responders. In a similar study, albeit with fewer samples, *Bacteroidales* were found enriched in patients who did not reach complete response post-treatment, and *Duodenibacillus massiliensis* was associated with a tumour response [144].

In summary, we have described potential biomarkers including molecular genetic markers, immunological markers and other biomarkers that have been analyzed from patients samples to predict the survival of rectal cancer patients and patients outcomes from chemoradiotherapy. In the next section, we will discuss cell-based models including commercial cancer cell lines and patient-derived xenografts/organoids originally from tumour cells of patients to predict tumour response to neoadjuvant chemoradiotherapy.

## Cell-based models to predict tumour response to neoadjuvant chemoradiotherapy

**Fig. 2:**
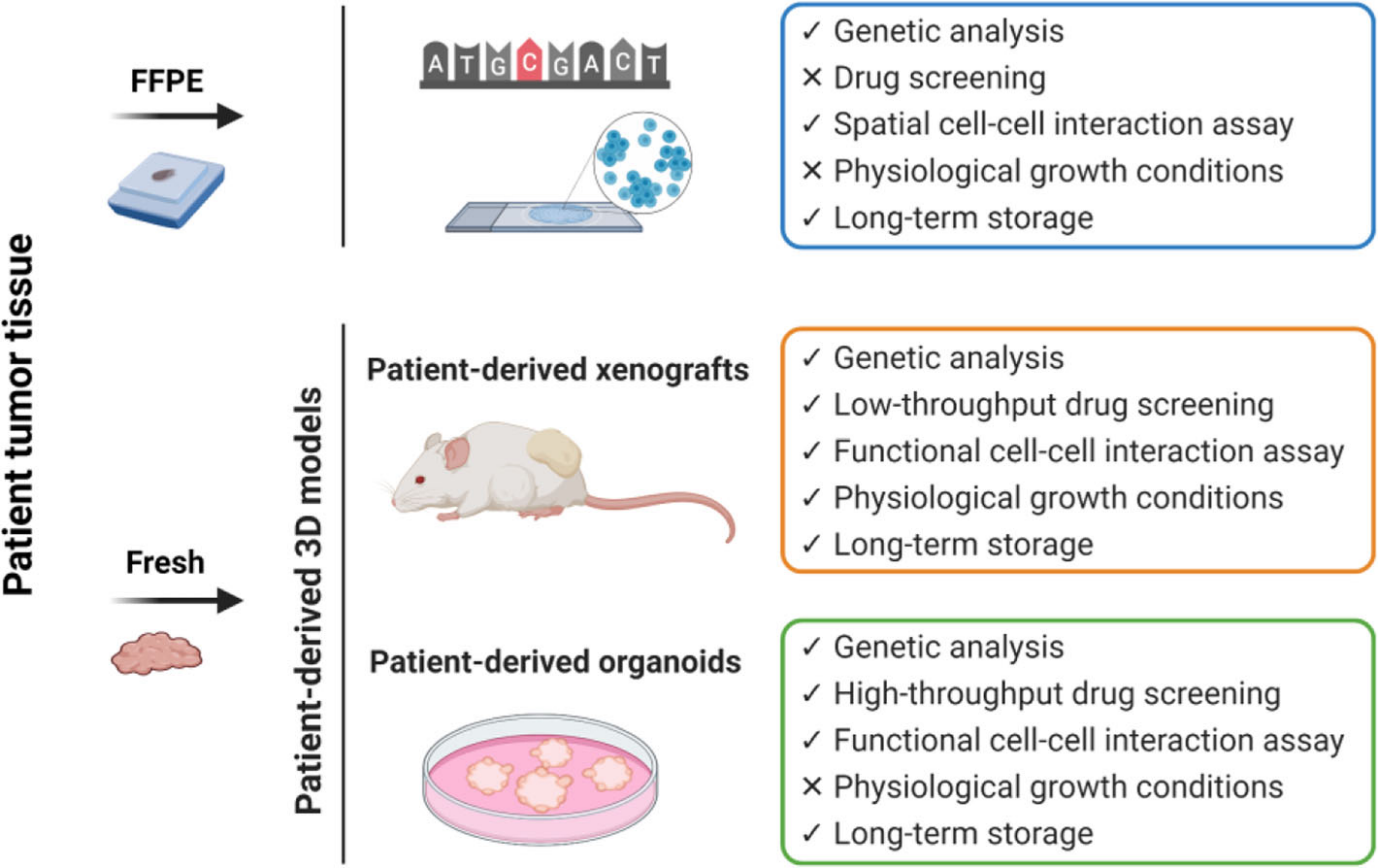
Advantages and disadvantages of formalin-fixed paraffin-embedded material, patient-derived xenografts and patient-derived organoids. These modalities differ in several aspects, including types of analysis that can be run, experimental conditions, and storage. FFPE: formalin-fixed paraffin-embedded

Immunohistochemical analyses of tumour biopsies have been used to enumerate cells in the tumour microenvironment with the aim of predicting effectiveness of therapies. Formalin-fixed paraffin-embedded (FFPE) tissues can be stored long-term and utilized for both immunohistochemical studies and for genetic analysis. However, although FFPE tissues can be useful in identifying new potential biomarkers, these tissues are not biologically active. Analysis of FFPE material is as such not applicable for gaining mechanistic insight into cellular interactions, unlike cell-based models (Fig. 2). Cell-based models, which have been used to mirror characteristics of tumour diseases, have also been used and their advantages and disadvantages are briefly described below.

### Cancer cell lines

Cancer cell lines kept in culture have been used for decades for in vitro modeling of cancer. The maintenance of cell lines grown in 2D cultures is fairly simple and genetic modifications can be performed using genome-editing techniques to knock genes of interest in or out [145]. In broad preclinical treatment prediction studies of colorectal cancer mainly colon cancer cell lines have been used. In a study using 77 colorectal cancer lines the response to 5-FU was assessed and found that mismatch repair deficiency correlated to 5-FU sensitivity [146]. A caveat of using cell lines is, however, that they often do not recapitulate the tumour disease well in terms of genomic alterations, protein expression, and therapeutic sensitivity [147–151]. In addition, cancer cell lines lack interactions with any surrounding tumour stroma. Therefore predicted sensitivity to drug treatments may not translate well into the clinical setting. Reasons for subpar recapitulation include genetic drift, artificial culture conditions and a lack of tumour microenvironment. To overcome these limitations, 3D cell culture platforms have been created to better mimic in vivo conditions (reviewed in [152, 153]). In many studies, these 3D culture platforms have proved more capable of inducing in vivo-like cell fates, and results from 3D studies demonstrate that increasing the dimensionality of the extracellular matrix from 2D to 3D can significantly impact proliferation, differentiation, cell survival and mechano-responses [154–157]. Thus 3D platforms are probably an attractive alternative for 2D cell culture.

### Patient-derived xenografts

With the aim of better recapitulating patient tumours and striving for personalized medicine, 3D culture platforms including patient-derived xenografts (PDX) and organoids have been developed. In the PDX approach, dissected tumour tissue from the patient is engrafted to severely immunocompromised mice [158]. Compared to cell lines, in vivo models more accurately recapitulate the histopathological and cellular structures of patient tumours. A study investigating the sensitivity of cetuximab in colorectal cancer PDX models found tumour responses exclusively in *KRAS* wild-type. This, and reported overall response rates in the study well resembles what is seen in the clinical setting [159]. In addition, it might be possible to retain not only the cellular structures including intra-tumoural and inter-tumoural heterogeneity but also the molecular and phenotypic characteristics of the original tumour in PDX models [160]. A few studies have assessed the relationship between colorectal cancer PDX models and clinical outcome. In one of these studies, 150 out of 241 transplanted colorectal tumours successfully engrafted and the tumourigenicity correlated with poor disease-free survival [161]. Moreover, PDX models harboring *KRAS* mutations showed resistance to anti-EGFR therapy, while PDXs without *KRAS* mutations responded to anti-EGFR therapy [159, 161]. In yet another study, 16 out of 18 metastatic colorectal cancer PDX models were successfully established (with 89.9% engraftment rate) and these recapitulated histological architecture of the original patient tumours [162]. However, generation of engrafted PDX models took approximately 50 days, which makes the use of the models for treatment guidance challenging. In summary, PDX models of colorectal cancer disease are encouraging as they recapitulate many features of the original tumours. However, they might have a relatively low rate of engraftment [163], and the development of colorectal cancer xenografts takes months, which increases costs of the model and impedes direct applicability for individualized treatments [164]. Another challenge is the slow take-rate that makes it difficult to use as predictive of treatment for the individual patient. In the future it is probable that PDX models may be used to predict treatment responses in groups of patients or for patients with metastastic disease.

### Patient-derived organoids

Patient-derived organoids are 3D cell culture systems where tumour cells from the patient tumour are expanded within a matrix. Organoids have been shown to largely recapitulate histopathological and cellular structures of the original tumour [165–167]. Though lacking the in vivo benefits of PDXs, patient-derived organoids instead come with lower costs, provide a platform for high-throughput applications and allow for accessible genetic manipulation [168, 169]. Applications have included therapy sensitivity prediction [169], studies of cancer cells microenvironment interactions [170], discovery of novel biomarkers and use in pre-clinical drug trials [171–174]. Patient-derived organoids from colorectal cancer could be generated and screened within 21 days potentially allowing direct clinical application [175]. The first establishment of a so-called “living biobank”, consisting of cultured patient-derived organoids and matching healthy organoids from patients with colorectal cancer, was reported in 2015 by van de Wetering and colleagues [176]. The 20 patient-derived colorectal tumour organoids recapitulated genetic alterations and gene expression profiles of the patient tumours. Proteomic profiles between tumour organoids derived from different patients were observed [177]. Patient-derived organoids from colorectal cancer also display marked intra-tumour mutational diversity [178]. In a recent study patient-derived rectal cancer organoids were achieved with a 77% success rate [179].

Several studies have demonstrated that patient-derived organoids can be used to study how various factors can affect therapy sensitivity. One study assembled a panel of patient-derived colorectal organoids including tumour organoids harbouring *RAS* mutations, tumour organoids and colon organoids with wild-type *RAS* as well as tumour organoids and colon organoids with CRISPR-introduced oncogenic *KRAS* mutations [171]. The presence of a mutation in *RAS* showed a correlation with resistance to RAS inhibitors. In another study, a colorectal cancer biobank comprising 35 patient-derived organoids and 59 patient-derived xenografts were used to identify novel biomarkers for prediction of sensitivity to the EGFR inhibitors [172]. Drug-screening results from patient-derived organoids derived from gastrointestinal cancers also matched that of corresponding patient tumours [173]. In this study, the patient-derived organoids were reported to have 93% specificity, 100% sensitivity, and 88% positive predictive value in predicting patient clinical response to the chemotherapy or targeted agents. In another study, optical metabolic imaging of colorectal cancer organoids were used for cell-level quantification of the response to treatment, thereby providing improved discrimination of therapeutic differences between the patient samples [174]. Establishment of CRC organoids appears to be more challenging from tumours that are characterized as MSI, *BRAF* mutated, poorly differentiated and/or of mucinous type [180], which may complicate the use as a tool for prediction.

Although there are several advantages of using patient-derived organoids there is still a need to improve the technique. Generated patient-derived organoids in published studies mostly lack fundamental extracellular elements and are grown in a highly artificial media, factors that could challenge clinical transition of findings [181].

The established organoids maintained the mutation status of actionable gene targets in corresponding tumour samples and recapitulated patient-specific clinical responses to radiation and chemotherapy. Tumouroids have been successfully transplanted orthotopically in vivo and both cancer-specific ex vivo and in vivo sensitivity to treatments could be assayed within 6–12 weeks after establishment [179]. A living organoid biobank from LARC patients treated with neoadjuvant chemoradiotherapy has also been generated [169]. Using this biobank it was shown that rectal cancer organoids resemble corresponding tumours both pathologically and genetically. Furthermore, response to chemoradiotherapy was matched with clinical data from patients with 92% specificity, 84% accuracy and 78% sensitivity. In another study with patient-derived xenografts and patient-derived organoids established from rectal cancer specimens prior to neoadjuvant therapy. Response to chemoradiotherapy was evaluated [182]. The histology of xenografted tumours correlated with corresponding rectal cancer tumours and had conserved mutational profiles. The patient-derived organoids recapitulated patient response to 5-FU and radiotherapy. Furthermore, xenografts and organoids harboring wild-type *KRAS* were more sensitive to cetuximab compared to those with mutated *KRAS* [182]. Another study similarly tested the sensitivity of rectal cancer organoids to 5-FU and oxaliplatin (FOLFOX) and compared it to clinical data [183]. This study saw that dMMR rectal cancer organoids had increased resistance to FOLFOX compared to pMMR organoids just like in the clinical setting.

Taken together it is probable that patient-derived organoids of rectal cancers could be a valuable translational research tool to discover novel biomarkers and personalized treatment strategies.

## Accommodating the tumour microenvironment in drug prediction studies

A potential consequence of mutations in cancer cells is the creation of neoantigens, which following display by MHC molecules on the surface of the tumour cell, can become visible to the immune system [184]. The recognition of these tumour neoantigens by T cells is of central importance for the efficiency of immunotherapies. To study these interactions and advance the field of cancer immunotherapy, access to tumour tissue and immune cells from the same patient could be of great use. Adding drug treatments in this setting could further aid in the study of tumour cell treatment resistance, as immune cells probably play an important role. Such studies can be conducted using, for example, PDX models with co-transferred autologous T cells or co-culturing tumour organoids with immune cells. In a recent study, tumour-specific T cell reactivity against dMMR colorectal cancer and non-small cell lung carcinoma patient-derived organoids was demonstrated [185]. Tumour-reactive T cells from peripheral blood of the patients were expanded in co-culture with patient-derived organoids. These T cells specifically recognized their autologous tumour organoids but not healthy organoids. An air-liquid patient-derived organoid culture system has also been developed [170]. These cultures recapitulated complex tumour architectures, including immune and stromal compartments. Single cell sequencing in this model showed that tumour-infiltrating T cell clones were closely conserved between the patient-derived organoids and their corresponding tumours. Moreover, patient-derived organoid cultures were shown to functionally recapitulate the response to PD1/PD-L1-dependent immune checkpoint inhibition. Although the immune compartment was initially preserved in these air-liquid cultures, the frequencies unfortunately waned and after 2 months the T cells were completely lost.

## Conclusions

Identifying biomarkers to predict the response of rectal tumours to neoadjuvant therapy is of great interest. As treatment responses vary drastically, such tools would aid patient care in the clinical setting. An abundance of various types of biomarkers have been evaluated, including many molecular genetic markers and immunological markers, several of which have been found correlating with clinical outcome. In addition, several types of cell-based models of rectal cancer have been developed and successfully used to predict sensitivity of tumour cells to treatment. Rectal cancer tumours are infiltrated by immune cells and have an active interplay with their microenvironment. This seems to play an important role both for the response to therapy and for long term survival of rectal cancers patients. Cell-based models can facilitate integration of both patient-specific tumour characteristics and the immune microenvironment, which has been utilized to predict treatment sensitivity and could potentially be used to further elucidate the mechanisms at play. Although a number of biomarkers and cell-based models have been shown to predict tumour response to treatment to various degrees, none are yet recommended for clinical use. The search for robust, novel methods to predict tumour sensitivity is on-going, and additional validation of identified markers is needed in order to facilitate clinical implementation.

## Data Availability

Not applicable.

## Abbreviations

5-FU: 5-fluorouracil
BRAF: V-raf murine sarcoma viral oncogene homolog B1
CCL5: C-C motif chemokine ligand 5
CEA: Carcinoembryonic antigen
cfDNA: Cell-free DNA
CRC: Colorectal cancer
CRP: C-reactive protein
CTCs: Circulating tumour cells
CTLA4: Cytotoxic T-lymphocyte-associated protein 4
dMMR: Mismatch repair deficient
EGF: Epidermal growth factor
EGFR: Epidermal growth factor receptor
EPCAM: Epithelial cellular adhesion molecule
ERBB2: Erb-B2 Receptor Tyrosine Kinase 2
ERBB3: Erb-B2 Receptor Tyrosine Kinase 3
ERK: Extracellular signal-regulated kinases
FFPE: Formalin-fixed paraffin-embedded
FOLFOX: Folonic acid + 5-FU + Oxaliplatin
FoxP3: Forkhead box P3
GPR1: G protein-coupled receptor 1
GrzB^+^: Granzyme B
IFN-γ: Interferon gamma
IGF2: Insulin-like growth factor 2
IL-6: Interleukin 6
INSIG1: Insulin-induced gene 1
ISB: Biopsy-adapted immunoscore
KRAS: Kirsten rat sarcoma
LARC: Locally advanced rectal cancer
lncRNA: long non-coding RNA
LNR: Lymph node ratio
MAPK: Mitogen-activated protein kinase
MGMT: O^6^-methylguanine-DNA-methyl-transferase
MHC: Major histocompatibility complex
miRNA: MicroRNA
MMR: Mismatch repair
MSI: Mismatch repair instable
MSI-H: Mismatch repair instable-high
mTOR: Mechanistic target of rapamycin
NF1: Neurofibromin 1
N/L ratio: Neutrophil-to-lymphocyte ratio
NRAS: Neuroblastoma RAS viral oncogene homolog
OBSL1: Obscurin-like 1
PD1: Programmed cell death protein 1
PD-L1: Programmed death-ligand 1
PDX: Patient-derived xenograft
PI3K: Phosphoinositide 3-kinase
PIK3CA: Phosphatidylinositol 3-kinase catalytic alpha polypeptide
PIK3R1: Phosphoinositide-3-kinase, regulatory subunit 1
P/L ratio: Platelet-to-lymphocyte ratio
pMMR: Mismatch repair proficient
PTEN: Phosphatase and tensin homolog
RAS: Rat sarcoma
TCF4: Transcription factor 4
TGF-β1: Transforming growth factor beta 1
TIL: Tumour-infiltrating lymphocyte
TIMP3: Tissue inhibitor of matrix metalloproteinases 3
TNF-α: Tumour necrosis factor alpha
TP53: Tumour protein p53
VEGF: Vascular endothelial growth factor
XIAP: X-linked inhibitor of apoptosis protein
